# Placental DNA methylation profile as predicting marker for autism spectrum disorder (ASD)

**DOI:** 10.1186/s10020-022-00593-3

**Published:** 2023-01-16

**Authors:** Amin Ravaei, Marco Emanuele, Giovanni Nazzaro, Luciano Fadiga, Michele Rubini

**Affiliations:** 1 Medical Genetics Laboratory, Department of Neuroscience and Rehabilitation, University of Ferrara, Via Fossato di Mortara 74, 44121 Ferrara, Italy; 2 Section of Physiology, Department of Neuroscience and Rehabilitation, University of Ferrara, Ferrara, Italy; 3 IIT@UniFe Center for Translational Neurophysiology of Speech and Communication (CTNSC), Istituto Italiano di Tecnologia, Ferrara, Italy

**Keywords:** Autism, Placenta, DNA methylation, Brain, Epigenetic

## Abstract

Autism spectrum disorder (ASD) is a neurodevelopmental disorder that impairs normal brain development and socio-cognitive abilities. The pathogenesis of this condition points out the involvement of genetic and environmental factors during in-utero life. Placenta, as an interface tissue between mother and fetus, provides developing fetus requirements and exposes it to maternal environment as well. Therefore, the alteration of DNA methylation as epigenetic consequence of gene-environmental interaction in the placenta could shed light on ASD pathogenesis. In this study, we reviewed the current findings on placental methylation status and its association with ASD. Differentially methylated regions (DMRs) in ASD-developing placenta were found to be mainly enriched in ASD gene loci affecting synaptogenesis, microtubule dynamics, neurogenesis and neuritogenesis. In addition, non-genic DMRs in ASD-placenta proposes an alternative contributing mechanism for ASD development. Our study highlights the importance of placental DNA methylation signature as a biomarker for ASD prediction.

## Background

**Fig. 1:**
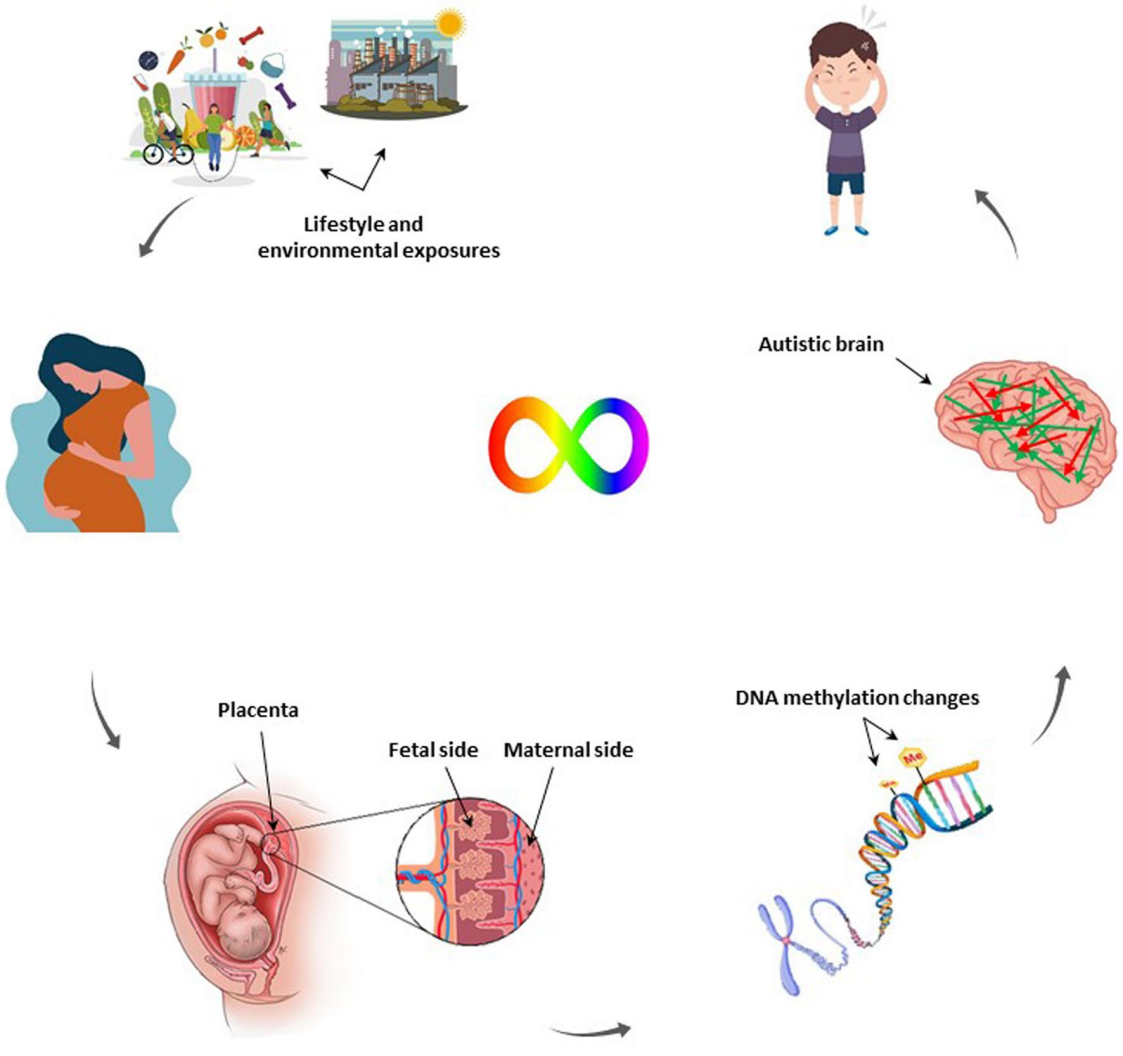
Placenta-brain axis. By affecting the in utero environment, maternal exposures could influence placental methylation and in turn alter gene expression, resulting in brain development impairments, possibly contributing to the ASD development. (The figure was designed using Vecteezy images; www.vecteezy.com)

Autism spectrum disorder (ASD) is a neurodevelopmental disorder characterized by persistent disturbances in social interaction and communication, restricted interests and repetitive behaviours (American Psychiatric Association 2013). This classic pattern of symptoms is also accompanied by distinctive impairments of cognitive and sensorimotor functions, including problems in perception (Happé and Frith 2006) and motor control (Cattaneo et al. 2007; Emanuele et al. 2021; Gowen and Hamilton 2013), as well as specific neurophysiological signatures (Oberman et al. 2016). ASD affects one in 54 children globally (Knopf 2020) and its pathogenesis is probably initiated during the in utero period, as supported by teratogen exposure timing (Strömland et al. 1994; Williams et al. 2001), anatomy of neurons (Bailey et al. 1998; Bauman and Kemper 2005; Rodier et al. 1996), and observed attitude differences during early childhood (Zwaigenbaum et al. 2005). Aetiology of ASD is a combination of genetic and environmental factors (Sandin et al. 2017; Tick et al. 2016). Several genome-wide studies have identified the inherited and de novo ASD risk factors (Autism Spectrum Disorders Working Group of The Psychiatric Genomics Consortium 2017). Environmental risk factors of ASD highlighted different in utero maternal exposures (Raz et al. 2015; Schmidt et al. 2012, 2011; Zerbo et al. 2013) including preconceptional and prenatal vitamin intake, such as B vitamin family which could reduce ASD risk by 40% if taken during the first month of pregnancy (Schmidt et al. 2012, 2011; Surén et al. 2013). Placenta, the temporary organ during pregnancy that develops shortly after implantation in the uterus and attaches to the wall of the uterus from which fetus’s umbilical cord arises (Turco and Moffett 2019), facilitates the exchange of nutrient, gas and waste between physically separate maternal and fetal circulations, passes immunity from mother to the fetus, and as an endocrine organ produces hormones (Gude et al. 2004), as well as neurotransmitters (Rosenfeld 2021). The placenta-derived hormones and neurotransmitters influence oxygen and nutrients transportation to the fetus, as well as brain development. The neurotransmitters such as serotonin, dopamine, norepinephrine/epinephrine and hormone like allopregnanolone produced by placenta affect several key aspects in brain development such as neurogenesis and neuronal migration (Handwerger and Freemark 2000; Rosenfeld 2021; Vacher et al. 2021). This influential correlation between the placenta and the brain is known as placenta-brain axis (Aziz et al. 2020; Santos Jr et al. 2020; Un Nisa et al. 2019) and led to the development of neuroplacentology field (Kratimenos and Penn 2019). Since genomic imprinting and the reprogramming of epigenetic modifications in the growing zygote are governed by the placentation process (Broad et al. 2016), neurodevelopmental disorders like ASD could be traced to placental disturbances (Rosenfeld 2021). This evidence is supported by several epidemiological and animal studies that have identified epigenetic modifications in the placenta correlated with neurodevelopmental pathologies (Meakin et al. 2018; Paquette et al. 2016; Rosenfeld 2020) (Fig. 1). Thus, we aimed to review the current findings on DNA methylation changes in the placenta and their association with ASD development.

## DNA methylation in ASD-placenta

DNA methylation status, as one of the principal epigenetic aspects, could reflect gene-environment interaction and play causal (Xu et al. 1999), consequential (Rodríguez-Ubreva et al. 2019) or intermediary (Liu et al. 2013) roles during pathogenesis. DNA methylation occurs by transferring a methyl group to the fifth carbon position of cytosine at cytosine–phosphate–guanine dinucleotides (CpG) by DNA methyltransferases (DNMTs) (Lin and Wang 2014). While CpGs are rare across the genome and mainly methylated (Jones 2012), they are clustered in the promoter region of genes, called CpG islands, and usually hypomethylated in transcriptionally active genes (van der Maarel 2008).

The human placenta has a distinct methylation profile found in all the three trimesters of pregnancy. It is characterized by large partially methylated domains (PMDs) (Schroeder et al. 2016) resembling oocytes and preimplantation embryos methylation, where methylation over gene bodies is positively associated with expression, (Zhu et al. 2022) and highly methylated domains (HMDs) (Schroeder et al. 2016, 2013) which is similar to the methylation pattern of fetal or adult tissues (Ali et al. 2014; Dekker and Sibai 2001; Mridha et al. 2017; Zhu et al. 2022). PMDs are mainly over 100 kb in length and make up 40% of the placental genome (Schmidt et al. 2016; Schroeder et al. 2013, 2011). Neuronal development and synaptic transmission genes, which are candidate loci for ASD, are enriched in the placental PMDs (Schroeder et al. 2016, 2011).

**Fig. 2:**
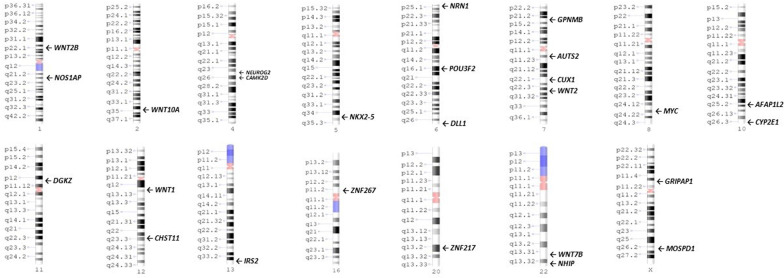
Chromosomal locations of most important differentially methylated genes in ASD-Placenta. The colors in the ideograms show: black and grey: Giemsa positive, red: centromere, light blue: variable region, and dark blue: stalk

Several genome-wide methylation studies identified thousands of significant differentially methylated CpGs belonging either to intergenic or intragenic regions (Bahado-Singh et al. 2021a; Bahado-Singh et al. 2021b; Bakulski et al. 2021; Santos Jr et al. 2020; Zhu et al. 2019). The intragenic CpGs have been reported involving some hundreds (Bakulski et al. 2021; Zhu et al. 2019) to more than four thousand genes (Bahado-Singh et al. 2021b) depending on the study design. These intragenic CpGs could be at Transcription Start Site (TSS) 200, TSS1500, 5′ UTR, 1st exon, gene body and 3′ UTR (Bahado-Singh et al. 2021a, 2021b; Schmidt et al. 2016; Schroeder et al. 2016; Zhu et al. 2022, 2019). The Simons Foundation Autism Research Initiative (SFARI) has identified and grouped the genes implicated in ASD susceptibility which are known as SFARI genes (Banerjee-Basu and Packer 2010). Differentially methylated regions (DMRs) in the ASD-placenta are dispersed throughout the genome and were reported in, but not limited to, SFARI genes (Bakulski et al. 2021). These DMRs, as identified by Ingenuity Pathways Analysis (IPA), affect different biological pathways mainly converging on synaptogenesis, microtubule dynamics, neurogenesis and neuritogenesis which finally influence neuron morphology, brain development and cognitive abilities (Bahado-Singh et al. 2021a, 2021b). The most emphasized differentially methylated genes reported in different studies with predictive value or as main player in a specific pathway were presented in Fig. 2 and Table 1.

**Table 1:** The most important differentially methylated genes in ASD-Placenta with predictive or pathways relevance value

Gene	Chromosome	Methylation	Product	Gene function	SFARI	Significance	Relevance	References
*NOS1AP*	1q23.3	Increased	Neuronal nitric oxide synthase 1 adaptor protein	Functions as an adapter protein connecting nNOS to specific targets like synapsins	N	GWS	Predictive	Bahado-Singh et al. (2021b)
*WNT2B*	1p13.2	Increased	Wnt Family Member 2B	Play a role in the canonical Wnt/beta-catenin signaling pathway	N	GWS	Microtubule dynamics pathway	Bahado-Singh et al. (2021b)
*WNT10A*	2q35	Decreased	Wnt Family Member 10A	Functions in the canonical Wnt/beta-catenin signaling pathway	N	GWS	Microtubule dynamics pathway	Bahado-Singh et al. (2021b)
*CAMK2D*	4q26	Increased	Calcium/Calmodulin-Dependent Protein Kinase Type II Delta Chain	Intracellular calcium signaling	N	GWS	Quantity of synapse pathway	Bahado-Singh et al. (2021b)
*NEUROG2*	4q25	Decreased	Neural-specific basic helix-loop-helix (bHLH) transcription factor	Specify progenitors to a neuronal fate on ectodermal cells and reprograms early postnatal astroglia to develop neurons	N	NS	Abnormal morphology of neurons pathway	Bahado-Singh et al. (2021b)
*NKX2-5*	5q35.1	Increased	Homeobox Protein Nkx-2.5 transcription factor	Functions in heart and the spleen development	N	GWS	Predictive	Bahado-Singh et al. (2021a)
*DLL1*	6q27	Increased/decreased	Encodes the Delta-like1 ligand of Notch receptors	Regulates neurogenesis, neurons differentiation, quiescence of neural stem cells and plays a role as a fate determinant during neural stem cells mitosis	Y	GWS	Predictive	Bahado-Singh et al. (2021b), Schroeder et al. (2016)
*POU3F2*	6q16.1	Decreased	POU Domain, Class 3, Transcription Factor 2	Neuronal differentiation and promoting the activation of corticotropin-releasing hormone regulated genes	N	NS	Abnormal morphology of neurons pathway	Bahado-Singh et al. (2021b)
*NRN1*	6p25.1	Increased	Neuritin	Promotes neurite outgrowth and arborisation during neuritogenesis	N	GWS	Predictive	Bahado-Singh et al. (2021a)
*AUTS2*	7q11.22	Decreased	Autism Susceptibility Gene 2 Protein	Contributes to dendrite and axon elongation and neuronal migration. It enhances rearrangement of the actin cytoskeleton, lamellipodia shaping and neurite elongation	Y	GWS	Neuritogenesis pathway	Bahado-Singh et al. (2021a), Bahado-Singh et al. (2021b)
*CUX1*	7q22.1	Increased	Homeobox Protein Cux-1	Encodes a transcription factor that controls neuronal differentiation in the brain, regulates dendrite development and branching, and dendritic spine formation in cortical layers II-III. It also play a controlling role during synaptogenesis	Y	GWS	Neuritogenesis pathway	Bahado-Singh et al. (2021b)
*WNT2*	7q31.2	Decreased	Wnt Family Member 2	Functions in the canonical Wnt/beta-catenin signaling pathway and likely involved in embryonic brain development by controlling the proliferation of dopaminergic precursors and neurons	N	GWS	Microtubule dynamics pathway	Bahado-Singh et al. (2021b)
*GPNMB*	7p15.3	Increased	Glycoprotein Nonmetastatic Melanoma Protein B	May be involved in growth delay and reduction of metastatic potential and could be a melanogenic enzyme	N	GWS	Predictive	Bahado-Singh et al. (2021a)
*MYC*	8q24.21	Decreased	Nuclear phosphoprotein (BHLH Transcription Factor)	Plays a role in cell cycle progression, apoptosis and cellular transformation	N	GWS	Microtubule dynamics pathway	Bahado-Singh et al. (2021b)
*CYP2E1*	10q26.3	Decreased	A member of cytochrome P450 superfamily	Contributes to catalysing reactions involved in drug metabolism and synthesis of cholesterol, steroids and other lipids Involved in the metabolism of drugs	N	GWS	Abnormal morphology of neurons pathway	Bahado-Singh et al. (2021a), Bahado-Singh et al. (2021b), Zhu et al. (2019)
*AFAP1L2*	10q25.3	Increased	Actin Filament-Associated Protein 1-Like 2	Plays several roles including positive regulation of epidermal growth factor receptor signaling pathway, regulation of gene expression; and controlling mitotic cell cycle	N	GWS	Predictive	Bahado-Singh et al. (2021b)
*DGKZ*	11p11.2	Decreased	Diacylglycerol Kinase Zeta	Reduces protein kinase C activity by controlling diacylglycerol levels in intracellular signaling cascade and signal transduction	N	NS only in maternal side	Predictive	Bahado-Singh et al. (2021b), Bakulski et al. (2021)
*CHST11*	12q23.3	NA	Carbohydrate Sulfotransferase 11	Catalyzes the transfer of sulfate to N-acetylgalactosamine (GalNAc) residue of chondroitin in cartilage and on the surfaces of cells and extracellular matrices	N	GWS in one CpG site, cg09418354	Predictive	Santos Jr et al. (2020)
*WNT1*	12q13.12	Decreased	Wnt Family Member 1	Functions in the canonical Wnt/beta-catenin signaling pathway and play a key role in the developing embryonic brain and central nervous system (CNS)	Y	GWS	Microtubule dynamics pathway	Bahado-Singh et al. (2021b)
*IRS2*	13q34	Increased/decreased	Encodes insulin receptor substrate 2	A cytoplasmic signalling molecule that mediates the influence of insulin and insulin-like growth factor 1 (IGF1) and cytokine receptors	N	GWS	Predictive	Bahado-Singh et al. (2021b), Zhu et al. (2019)
*ZNF267*	16p11.2	Increased	Zinc Finger Protein 267	Activates DNA-binding transcription activator function, RNA polymerase II-specific and RNA polymerase II cis-regulatory region sequence-specific DNA binding activity	N	GWS	Predictive	Bahado-Singh et al. (2021a)
*ZNF217*	20q13.2	Increased	Zinc Finger Protein 217	Activates DNA-binding transcription repressor function, RNA polymerase II-specific and RNA polymerase II cis-regulatory region sequence-specific DNA binding activity and regulates neuron-specific genes such as *CoREST* and *HDAC2*	N	GWS	Predictive	Bahado-Singh et al. (2021a)
*NHIP*	22q13.33	Decreased	lncRNA	Hypoxia responsive regulatory gene	N	GWS	Predictive	Zhu et al. (2022)
*WNT7B*	22q13.31	Increased	Wnt Family Member 7B	Functions in the canonical Wnt/beta-catenin signaling pathway and is necessary for central nervous system (CNS) angiogenesis and blood–brain barrier regulation	N	GWS	Microtubule dynamics pathway	Bahado-Singh et al. (2021b)
*GRIPAP1*	Xp11.23	Increased	GRIP1 Associated Protein 1	Involved in neuronal cytoskeleton organization	N	GWS	Predictive	Bahado-Singh et al. (2021b)
*MOSPD1*	Xq26.3	Increased	Motile Sperm Domain Containing 1	Mesenchymal stem cells (MSCs) proliferation and differentiation	N	GWS	Predictive	Bahado-Singh et al. (2021b)

*GWS* genome-wide significant, *NS* nominally significant, *NA* not available, *Y* yes, *N* no

### Influential differentially methylated genes in ASD-placenta affecting brain development

*NOS1AP* is one of the hypermethylated genes in the placenta of ASD (Bahado-Singh et al. 2021b). The product of this gene (NOS1AP) is a cytosolic protein that binds to the signalling molecule, neuronal nitric oxide synthase (nNOS). NOS1AP, as an adapter protein, links nNOS to specific targets, such as synapsins, whose function is necessary at a presynaptic level (Majmundar et al. 2021). *CAMK2D* is another hypermethylated gene in the placenta of ASD (Bahado-Singh et al. 2021b). Since the product of this gene belongs to the serine/threonine protein kinase family and Ca^2+^/calmodulin-dependent protein kinase subfamily, its dysregulation could affect intracellular calcium signalling which is crucial for several aspects of plasticity at glutamatergic synapses (Abraham et al. 2019; Martinez-Pena y Valenzuela et al. 2010). *AUTS2* gene was found to be hypermethylated in ASD-placenta (Bahado-Singh et al. 2021b). During embryonic brain development, it contributes to dendrite and axon elongation and neuronal migration. It enhances rearrangement of the actin cytoskeleton, lamellipodia shaping and neurite elongation (Gao et al. 2014). *CUX1* gene encodes transcription factor Homeobox Protein Cux-1 and is hypermethylated in the placenta (Bahado-Singh et al. 2021b). It is known that CUX1 regulates neuronal differentiation in the brain, development and branching of dendrite, and formation of dendritic spines in cortical layers II-III. It also plays a controlling role during synaptogenesis (Cubelos et al. 2010). *NEUROG2* gene that is usually expressed in neural progenitor cells within the developing central and peripheral nervous systems (Aravantinou-Fatorou et al. 2022) was found to be hypomethylated in ASD (Bahado-Singh et al. 2021b). This gene encodes a neural-specific basic helix-loop-helix (bHLH) transcription factor which could determine a neuronal fate on ectodermal cells within developing brain and functions in the differentiation and survival of midbrain dopaminergic neurons (Aravantinou-Fatorou et al. 2022; Park et al. 2008). *NEUROG2* induces excitatory neurons in human cortices, and its knockout results in lack of excitatory neurons demonstrating its key function in ASD (Deneault et al. 2018; Nehme et al. 2018). *NRN1* is another hypermetylated gene in ASD-placenta (Bahado-Singh et al. 2021a) which is expressed in postmitotic-differentiating neurons of the developing nervous system and neuronal structures. *NRN1* by encoding a member of the neuritin family contributes to neurite outgrowth and arborization, demonstrating its function in promoting neuritogenesis. Overexpression of *NRN1* could be correlated with astrocytoma progression (Nedivi et al. 1993; Zhang et al. 2011). *POU3F2,* another hypomethylated gene, encodes a transcription factor that is involved in the process of neuronal differentiation and promotes the activation of corticotropin-releasing hormone regulated genes (Lin et al. 2018). This gene has high expression in the developing brain and is considered as a master regulator of downstream ASD candidate genes (Bahado-Singh et al. 2021b; Lin et al. 2018). *GRIPAP1* gene that encodes a guanine nucleotide exchange factor for the Ras family of small G proteins (RasGEF) was found to be hypermethylated in ASD (Bahado-Singh et al. 2021b). The encoded protein, by regulating the endosomal recycling back to the neuronal plasma membrane within dendritic spines, functions in the maintenance of dendritic spine morphology. Its activity is necessary for recycling α-amino-3-hydroxy-5-methyl-4-isoxazolepropionic acid (AMPA) receptor to dendrite membranes and synaptic plasticity (Hoogenraad et al. 2010). Members of *WNT* family gene including *WNT1, WNT2, WNT2B, WNT7B* and *WNT10A* had altered methylation levels in ASD placenta (Bahado-Singh et al. 2021b). The WNT signalling pathway governs multiple processes, including embryonic development and tissue homeostasis (Bae and Hong 2018). *WNT1, WNT2* and *WNT10A* genes are hypomethylated while *WNT2B* and *WNT7B* are hypermethylated in ASD-placenta (Bahado-Singh et al. 2021b). *WNT1*, a very conserved gene in evolution encoding a protein 98% identical to the mouse Wnt1 protein, is known to play a key role in the developing embryonic brain and central nervous system (CNS), specifically for the induction of the mesencephalon and cerebellum (Guo et al. 2007; Lekven et al. 2019; Pieters et al. 2020). *WNT2* is probably involved in embryonic brain development by regulating the proliferation of dopaminergic precursors and neurons (Sousa et al. 2010). *WNT7B* plays a role in central nervous system (CNS) angiogenesis and blood–brain barrier regulation (Eubelen et al. 2018; Eubelen et al. 2018) *DLL1* gene that encodes the Delta-like1 ligand of Notch receptors was found to be hypermethylated in the placenta of ASD (Schroeder et al. 2016). *DLL1* regulates neurogenesis, neurons differentiation, quiescence of neural stem cells and plays a role as a fate determinant during neural stem cells mitosis. It influences brain development at different levels including neocortex development, cerebellar development by regulating Bergmann glial monolayer formation and its morphological maturation, spinal cord development by regulating neurogenesis through preventing the premature differentiation of neural progenitors and maintaining progenitors in spinal cord (Barton and Fendrik 2013; Hiraoka et al. 2013; Nelson et al. 2013; Ramos et al. 2010; Solecki et al. 2001). Protocadherin (Pcdh) is a gene family functioning in the formation of neural networking and synaptogenesis (Peek et al. 2017). Several loci of *PCDH* gene family were found to be hypomethylated in ASD placenta likely affecting the quantity of synapse (Bahado-Singh et al. 2021b).

### Non-genic DNA methylation pattern in ASD-placenta

The genomic localizations of methylation sites are classified according to the distance from CpG islands known as: (a) shores: regions up to 2 kb from CpG island, (b) shelves: regions from 2 to 4 kb from CpG island and (c) open sea: the rest of the genome (Sandoval et al. 2011). In the fetal side of the placenta, the most powerful associations with global methylation were reported in the shelf and open sea, regions which are not necessarily connected to specific genes (Bakulski et al. 2021). In addition, the observation of different methylation patterns in thousands of intergenic CpGs in ASD placenta reported in other studies (Bahado-Singh et al. 2021a, 2021b) supports that non-genic mechanisms contribute to ASD development (Bakulski et al. 2021). It is known that aberrant methylation in intergenic regions is associated with histone methylation and euchromatin modification which could influence the reprogramming of the 3D organization of chromatin and activation of distal enhancers (Li et al. 2021). This observation could be further supported by the presence of loss-of-function variants in ASD risk genes such as *BAF* (Lo et al. 2022), *CHD8* (De Rubeis et al. 2014) and *SETD5* (Nakagawa et al. 2020; Sessa et al. 2019) which function is involved in chromatin remodelling and are affecting multiple cellular processes such as transcription and replication.

## Conclusion and future direction

As the pathology of ASD is mostly limited to the development of the brain, the most appropriate approach to investigate DNA methylation in this condition should be through samples of brain tissue. However, analysing brain tissue is challenging due to small sample size, limited replication capacity, timing of collection after disease onset and aging (Bakulski et al. 2016). Although lacking the same target tissues of the brain, investigating perinatal tissues has some privileges, including larger sample size and timing prior to disease manifestation (Bakulski et al. 2016). Bakulski et al., compared placenta DNA methylation level with other peripheral tissues including early- and late-pregnancy maternal blood and infant cord blood and tested them for enrichment in ASD genetic loci including 881 SFARI genes. They evidenced that among 839 enriched SFARI genes, placenta showed enrichment for more than 400 genes among which 144 genes overlapped in all tissue types, which implies reliability of placenta for ASD methylation studies (Bakulski et al. 2021). However, several aspects should be considered in studying the methylation of placenta in ASD. First, methylation of placenta is less likely to be a pathological consequence and more likely to be an intermediate phase in ASD process (Bakulski et al. 2021). Second, placenta is a heterogeneous mixture of cells such as trophoblasts mesenchymal stromal cells, fetal vascular and hematopoetic cells (Schmidt et al. 2016), which have different gene expression patterns and correspondingly would have different methylation signature. Third, placenta is a unique tissue featuring the juncture of two separate genomes, i.e., mother and fetus, that could accordingly have differences in their epigenetic machinery. In large scale level, no distinguishable methylation differences between maternal and fetal side (Schroeder et al. 2013) or cell type (Schroeder et al. 2015) have been observed (Schmidt et al. 2016) but nominally significant differences between maternal and fetus side have been reported (Bakulski et al. 2021). Last, similar to reported differences in DNA methylation in umbilical cord tissue from preterm and full-term pregnancies (Wu et al. 2019), placenta from preterm ASD (Bahado-Singh et al. 2021a) and full-term ASD (Bahado-Singh et al. 2021b) may have differences in their methylation profiles.

The placenta, as an accessible tissue with distinctive global and site-specific DNA methylation profiles, could provide important information about ASD development as it is a more precise catalogue of obstetric, perinatal and labor influences than other tissues and could have precise diagnostic value, however, following confirmation of the available predictive evidence by replication studies with larger sample size as most of the current studies suffer from limited sample size or being underpowered. In addition, there are heterogeneity in study design of the available reports which limits providing top differentially methylated loci as a few of them such as *DDL1*, *AUTS2*, *CYP2E1*, and *IRS2* have been emphasized in more than one study. In perspective, aggregating additional datasets such as mRNA and miRNA expression with placental DNA methylation data (Santos Jr et al. 2020), using an extra unbiased tool such as whole genome bisulfite sequencing (WGBA), which has identified the novel locus *NHIP* that had been missed by standard array-based methods (Zhu et al. 2022), and applying effective AI algorithms achieving a highly accurate prediction of ASD (Bahado-Singh et al. 2021a, 2021b) could establish a fine-tuned diagnostic pipeline. In conclusion, the available pieces of evidence support that the methylation changes in the placenta might be a relevant informative biomarker for ASD prediction.

## Data Availability

Not applicable.

## Abbreviations

ASD: Autism spectrum disorder
MDRs: Differentially methylated regions
CpG: Cytosine–phosphate–guanine dinucleotides
DNMTs: DNA methyltransferases
PMDs: Partially methylated domains
HMDs: Highly methylated domains
TSS: Transcription start site
SFARI: Simons Foundation Autism Research Initiative
IPA: Ingenuity pathways analysis
nNOS: Neuronal nitric oxide synthase
bHLH: Basic helix-loop-helix
RasGEF: Ras family of small G proteins
AMPA: α-Amino-3-hydroxy-5-methyl-4-isoxazolepropionic acid
CNS: Central nervous system
Pcdh: Protocadherin
WGBA: Whole genome bisulfite sequencing

## References

[CR1] Abraham JR, Szoko N, Barnard J, Rubin RA, Schlatzer D, Lundberg K (2019). Proteomic investigations of autism brain identify known and novel pathogenetic processes. Sci Rep.

[CR2] Ali F, Thaver I, Khan SA (2014). Assessment of dietary diversity and nutritional status of pregnant women in Islamabad, Pakistan. J Ayub Med Coll Abbottabad.

[CR3] Aravantinou-Fatorou K, Vejdani S, Thomaidou D (2022). Cend1 and Neurog2 efficiently reprogram human cortical astrocytes to neural precursor cells and induced-neurons. Int J Dev Biol.

[CR4] American Psychiatric Association. DSM 5. Diagnostic and statistical manual of mental disorders (5th ed.). 2013.

[CR5] Autism Spectrum Disorders Working Group of The Psychiatric Genomics Consortium (2017). Meta-analysis of GWAS of over 16,000 individuals with autism spectrum disorder highlights a novel locus at 10q2432 and a significant overlap with schizophrenia. Mol Autism..

[CR6] Aziz A, Saleem S, Nolen TL, Pradhan NA, McClure EM, Jessani S (2020). Why are the Pakistani maternal, fetal and newborn outcomes so poor compared to other low and middle-income countries?. Reprod Health.

[CR7] Bae SM, Hong JY (2018). The Wnt signaling pathway and related therapeutic drugs in autism spectrum disorder. Clin Psychopharmacol Neurosci.

[CR8] Bahado-Singh RO, Vishweswaraiah S, Aydas B, Radhakrishna U (2021). Artificial intelligence and placental DNA methylation: newborn prediction and molecular mechanisms of autism in preterm children. J Matern Fetal Neonatal Med.

[CR9] Bahado-Singh RO, Vishweswaraiah S, Aydas B, Radhakrishna U (2021). Placental DNA methylation changes and the early prediction of autism in full-term newborns. PLoS ONE.

[CR10] Bailey A, Luthert P, Dean A, Harding B, Janota I, Montgomery M (1998). A clinicopathological study of autism. Brain.

[CR11] Bakulski KM, Halladay A, Hu VW, Mill J, Fallin MD (2016). Epigenetic research in neuropsychiatric disorders: the ‘tissue issue’. Curr Behav Neurosci Rep.

[CR12] Bakulski KM, Dou JF, Feinberg JI, Aung MT, Ladd-Acosta C, Volk HE (2021). Autism-associated DNA methylation at birth from multiple tissues is enriched for autism genes in the early autism risk longitudinal investigation. Front Mol Neurosci.

[CR13] Banerjee-Basu S, Packer A (2010). SFARI Gene: an evolving database for the autism research community. Dis Model Mech.

[CR14] Barton A, Fendrik AJ (2013). Sustained vs. oscillating expressions of Ngn2, Dll1 and Hes1: a model of neural differentiation of embryonic telencephalon. J Theor Biol.

[CR15] Bauman ML, Kemper TL (2005). Neuroanatomic observations of the brain in autism: a review and future directions. Int J Dev Neurosci.

[CR16] Broad KD, Rocha-Ferreira E, Hristova M (2016). Placental, matrilineal, and epigenetic mechanisms promoting environmentally adaptive development of the mammalian brain. Neural Plast.

[CR17] Cattaneo L, Fabbri-Destro M, Boria S, Pieraccini C, Monti A, Cossu G (2007). Impairment of actions chains in autism and its possible role in intention understanding. Proc Natl Acad Sci U S A.

[CR18] Cubelos B, Sebastián-Serrano A, Beccari L, Calcagnotto ME, Cisneros E, Kim S (2010). Cux1 and Cux2 regulate dendritic branching, spine morphology, and synapses of the upper layer neurons of the cortex. Neuron.

[CR19] De Rubeis S, He X, Goldberg AP, Poultney CS, Samocha K, Cicek AE (2014). Synaptic, transcriptional and chromatin genes disrupted in autism. Nature.

[CR20] Dekker G, Sibai B (2001). Primary, secondary, and tertiary prevention of pre-eclampsia. Lancet.

[CR21] Deneault E, White SH, Rodrigues DC, Ross PJ, Faheem M, Zaslavsky K (2018). Complete disruption of autism-susceptibility genes by gene editing predominantly reduces functional connectivity of isogenic human neurons. Stem Cell Reports.

[CR22] Emanuele M, Nazzaro G, Marini M, Veronesi C, Boni S, Polletta G (2021). Motor synergies: evidence for a novel motor signature in autism spectrum disorder. Cognition.

[CR23] Eubelen M, Bostaille N, Cabochette P, Gauquier A, Tebabi P, Dumitru AC (2018). A molecular mechanism for Wnt ligand-specific signaling. Science.

[CR24] Gao Z, Lee P, Stafford JM, von Schimmelmann M, Schaefer A, Reinberg D (2014). An AUTS2-Polycomb complex activates gene expression in the CNS. Nature.

[CR25] Gowen E, Hamilton A. Motor abilities in autism: a review using a computational context. 2013;43(2):323–44. 10.1007/s10803-012-1574-0 22723127

[CR26] Gude NM, Roberts CT, Kalionis B, King RG (2004). Growth and function of the normal human placenta. Thromb Res.

[CR27] Guo C, Qiu H-Y, Huang Y, Chen H, Yang R-Q, Chen S-D (2007). Lmx1b is essential for Fgf8 and Wnt1 expression in the isthmic organizer during tectum and cerebellum development in mice. Development.

[CR28] Handwerger S, Freemark M (2000). The roles of placental growth hormone and placental lactogen in the regulation of human fetal growth and development. J Pediatr Endocrinol Metab.

[CR29] Happé F, Frith U (2006). The weak coherence account: detail-focused cognitive style in autism spectrum disorders. J Autism Dev Disord.

[CR30] Hiraoka Y, Komine O, Nagaoka M, Bai N, Hozumi K, Tanaka K (2013). Delta-like 1 regulates Bergmann glial monolayer formation during cerebellar development. Mol Brain.

[CR31] Hoogenraad CC, Popa I, Futai K, Martinez-Sanchez E, Sanchez-Martinez E, Wulf PS (2010). Neuron specific Rab4 effector GRASP-1 coordinates membrane specialization and maturation of recycling endosomes. PLoS Biol.

[CR32] Jones PA (2012). Functions of DNA methylation: islands, start sites, gene bodies and beyond. Nat Rev Genet.

[CR33] Knopf A (2020). Autism prevalence increases from 1 in 60 to 1 in 54: CDC. Brown Univ Child Adolesc Psychopharmacol Update.

[CR34] Kratimenos P, Penn AA (2019). Placental programming of neuropsychiatric disease. Pediatr Res.

[CR35] Lekven AC, Lilie CJ, Gibbs HC, Green DG, Singh A, Yeh AT (2019). Analysis of the wnt1 regulatory chromosomal landscape. Dev Genes Evol.

[CR36] Li Y, Chen X, Lu C (2021). The interplay between DNA and histone methylation: molecular mechanisms and disease implications. EMBO Rep.

[CR37] Lin R-K, Wang Y-C (2014). Dysregulated transcriptional and post-translational control of DNA methyltransferases in cancer. Cell Biosci.

[CR38] Lin Y-MJ, Hsin I-L, Sun HS, Lin S, Lai Y-L, Chen H-Y (2018). NTF3 is a novel target gene of the transcription factor POU3F2 and is required for neuronal differentiation. Mol Neurobiol.

[CR39] Liu Y, Aryee MJ, Padyukov L, Fallin MD, Hesselberg E, Runarsson A (2013). Epigenome-wide association data implicate DNA methylation as an intermediary of genetic risk in rheumatoid arthritis. Nat Biotechnol.

[CR40] Lo T, Kushima I, Aleksic B, Kato H, Nawa Y, Hayashi Y (2022). Sequencing of selected chromatin remodelling genes reveals increased burden of rare missense variants in ASD patients from the Japanese population. Int Rev Psychiatry.

[CR41] Majmundar AJ, Buerger F, Forbes TA, Klämbt V, Schneider R, Deutsch K (2021). Recessive NOS1AP variants impair actin remodeling and cause glomerulopathy in humans and mice. Sci Adv.

[CR42] Martinez-Pena y Valenzuela I, Mouslim C, Akaaboune M (2010). Calcium/calmodulin kinase II-dependent acetylcholine receptor cycling at the mammalian neuromuscular junction in vivo. J Neurosci.

[CR43] Meakin CJ, Martin EM, Santos HP, Mokrova I, Kuban K, O’Shea TM (2018). Placental CpG methylation of HPA-axis genes is associated with cognitive impairment at age 10 among children born extremely preterm. Horm Behav.

[CR44] Mridha MK, Matias SL, Paul RR, Hussain S, Sarker M, Hossain M (2017). Prenatal lipid-based nutrient supplements do not affect pregnancy or childbirth complications or cesarean delivery in Bangladesh: a cluster-randomized controlled effectiveness trial. J Nutr.

[CR45] Nakagawa T, Hattori S, Nobuta R, Kimura R, Nakagawa M, Matsumoto M (2020). The autism-related protein SETD5 controls neural cell proliferation through epigenetic regulation of rDNA expression. J iScience..

[CR46] Nedivi E, Hevroni D, Naot D, Israeli D, Citri Y (1993). Numerous candidate plasticity-related genes revealed by differential cDNA cloning. Nature.

[CR47] Nehme R, Zuccaro E, Ghosh SD, Li C, Sherwood JL, Pietilainen O (2018). Combining NGN2 programming with developmental patterning generates human excitatory neurons with NMDAR-mediated synaptic transmission. Cell Rep.

[CR48] Nelson BR, Hodge RD, Bedogni F, Hevner RF (2013). Dynamic interactions between intermediate neurogenic progenitors and radial glia in embryonic mouse neocortex: potential role in Dll1-Notch signaling. J Neurosci.

[CR49] Oberman LM, Enticott PG, Casanova MF, Rotenberg A, Pascual-Leone A, McCracken JT (2016). Transcranial magnetic stimulation in autism spectrum disorder: challenges, promise, and roadmap for future research. Autism Res.

[CR50] Paquette AG, Houseman EA, Green BB, Lesseur C, Armstrong DA, Lester B (2016). Regions of variable DNA methylation in human placenta associated with newborn neurobehavior. Epigenetics.

[CR51] Park C-H, Kang JS, Yoon E-H, Shim J-W, Suh-Kim H, Lee S-H (2008). Proneural bHLH neurogenin 2 differentially regulates Nurr1-induced dopamine neuron differentiation in rat and mouse neural precursor cells in vitro. FEBS Lett.

[CR52] Peek SL, Mah KM, Weiner JA (2017). Regulation of neural circuit formation by protocadherins. Cell Mol Life Sci.

[CR53] Pieters T, Sanders E, Tian H, van Hengel J, van Roy F (2020). Neural defects caused by total and Wnt1-Cre mediated ablation of p120ctn in mice. BMC Dev Biol.

[CR54] Ramos C, Rocha S, Gaspar C, Henrique D (2010). Two Notch ligands, Dll1 and Jag1, are differently restricted in their range of action to control neurogenesis in the mammalian spinal cord. PLoS ONE.

[CR55] Raz R, Roberts AL, Lyall K, Hart JE, Just AC, Laden F (2015). Autism spectrum disorder and particulate matter air pollution before, during, and after pregnancy: a nested case-control analysis within the Nurses’ Health Study II Cohort. Environ Health Perspect.

[CR56] Rodier PM, Ingram JL, Tisdale B, Nelson S, Romano J (1996). Embryological origin for autism: developmental anomalies of the cranial nerve motor nuclei. J Comp Neurol.

[CR57] Rodríguez-Ubreva J, de la Calle-Fabregat C, Li T, Ciudad L, Ballestar ML, Català-Moll F (2019). Inflammatory cytokines shape a changing DNA methylome in monocytes mirroring disease activity in rheumatoid arthritis. Ann Rheum Dis.

[CR58] Rosenfeld CS (2020). Placental serotonin signaling, pregnancy outcomes, and regulation of fetal brain development†. Biol Reprod.

[CR59] Rosenfeld CS (2021). The placenta-brain-axis. J Neurosci Res.

[CR60] Sandin S, Lichtenstein P, Kuja-Halkola R, Hultman C, Larsson H, Reichenberg A (2017). The heritability of autism spectrum disorder. JAMA.

[CR61] Sandoval J, Heyn H, Moran S, Serra-Musach J, Pujana MA, Bibikova M (2011). Validation of a DNA methylation microarray for 450,000 CpG sites in the human genome. Epigenetics.

[CR62] Santos HP, Bhattacharya A, Joseph RM, Smeester L, Kuban KCK, Marsit CJ (2020). Evidence for the placenta-brain axis: multi-omic kernel aggregation predicts intellectual and social impairment in children born extremely preterm. Mol Autism.

[CR63] Schmidt RJ, Hansen RL, Hartiala J, Allayee H, Schmidt LC, Tancredi DJ (2011). Prenatal vitamins, one-carbon metabolism gene variants, and risk for autism. Epidemiology.

[CR64] Schmidt RJ, Tancredi DJ, Ozonoff S, Hansen RL, Hartiala J, Allayee H (2012). Maternal periconceptional folic acid intake and risk of autism spectrum disorders and developmental delay in the CHARGE (CHildhood Autism Risks from Genetics and Environment) case-control study. Am J Clin Nutr.

[CR65] Schmidt RJ, Schroeder DI, Crary-Dooley FK, Barkoski JM, Tancredi DJ, Walker CK (2016). Self-reported pregnancy exposures and placental DNA methylation in the MARBLES prospective autism sibling study. Environ Epigenet..

[CR66] Schroeder DI, Lott P, Korf I, LaSalle JM (2011). Large-scale methylation domains mark a functional subset of neuronally expressed genes. Genome Res.

[CR67] Schroeder DI, Blair JD, Lott P, Yu HOK, Hong D, Crary F (2013). The human placenta methylome. Proc Natl Acad Sci U S A.

[CR68] Schroeder DI, Jayashankar K, Douglas KC, Thirkill TL, York D, Dickinson PJ (2015). Early Developmental and Evolutionary Origins of Gene Body DNA Methylation Patterns in Mammalian Placentas. PLoS Genet.

[CR69] Schroeder DI, Schmidt RJ, Crary-Dooley FK, Walker CK, Ozonoff S, Tancredi DJ (2016). Placental methylome analysis from a prospective autism study. Mol Autism.

[CR70] Sessa A, Fagnocchi L, Mastrototaro G, Massimino L, Zaghi M, Indrigo M (2019). SETD5 regulates chromatin methylation state and preserves global transcriptional fidelity during brain development and neuronal wiring. Neuron.

[CR71] Solecki DJ, Liu XL, Tomoda T, Fang Y, Hatten ME (2001). Activated Notch2 signaling inhibits differentiation of cerebellar granule neuron precursors by maintaining proliferation. Neuron.

[CR72] Sousa KM, Villaescusa JC, Cajanek L, Ondr JK, Castelo-Branco G, Hofstra W (2010). Wnt2 regulates progenitor proliferation in the developing ventral midbrain. J Biol Chem.

[CR73] Strömland K, Nordin V, Miller M, Akerström B, Gillberg C (1994). Autism in thalidomide embryopathy: a population study. Dev Med Child Neurol.

[CR74] Surén P, Roth C, Bresnahan M, Haugen M, Hornig M, Hirtz D (2013). Association between maternal use of folic acid supplements and risk of autism spectrum disorders in children. JAMA.

[CR75] Tick B, Bolton P, Happé F, Rutter M, Rijsdijk F (2016). Heritability of autism spectrum disorders: a meta-analysis of twin studies. J Child Psychol Psychiatry.

[CR76] Turco MY, Moffett A (2019). Development of the human placenta. Development.

[CR77] Un Nisa S, Shaikh AA, Kumar R (2019). Maternal and Fetal Outcomes of Pregnancy-related Hypertensive Disorders in a Tertiary Care Hospital in Sukkur, Pakistan. Cureus.

[CR78] Vacher C-M, Lacaille H, O’Reilly JJ, Salzbank J, Bakalar D, Sebaoui S (2021). Placental endocrine function shapes cerebellar development and social behavior. Nat Neurosci.

[CR79] van der Maarel SM (2008). Epigenetic mechanisms in health and disease. Ann Rheum Dis.

[CR80] Williams G, King J, Cunningham M, Stephan M, Kerr B, Hersh JH (2001). Fetal valproate syndrome and autism: additional evidence of an association. Dev Med Child Neurol.

[CR81] Wu Y, Lin X, Lim IY, Chen L, Teh AL, MacIsaac JL (2019). Analysis of two birth tissues provides new insights into the epigenetic landscape of neonates born preterm. Clin Epigenetics.

[CR82] Xu GL, Bestor TH, Bourc’his D, Hsieh CL, Tommerup N, Bugge M (1999). Chromosome instability and immunodeficiency syndrome caused by mutations in a DNA methyltransferase gene. Nature.

[CR83] Zerbo O, Iosif A-M, Walker C, Ozonoff S, Hansen RL, Hertz-Picciotto I (2013). Is maternal influenza or fever during pregnancy associated with autism or developmental delays? Results from the CHARGE (CHildhood Autism Risks from Genetics and Environment) study. J Autism Dev Disord.

[CR84] Zhang L, Zhao Y, Wang C, Fei Z, Wang Y, Li L (2011). Neuritin expression and its relation with proliferation, apoptosis, and angiogenesis in human astrocytoma. Med Oncol.

[CR85] Zhu Y, Mordaunt CE, Yasui DH, Marathe R, Coulson RL, Dunaway KW (2019). Placental DNA methylation levels at CYP2E1 and IRS2 are associated with child outcome in a prospective autism study. Hum Mol Genet.

[CR86] Zhu Y, Gomez JA, Laufer BI, Mordaunt CE, Mouat JS, Soto DC (2022). Placental methylome reveals a 22q13.33 brain regulatory gene locus associated with autism. Genome Biol.

[CR87] Zwaigenbaum L, Bryson S, Rogers T, Roberts W, Brian J, Szatmari P (2005). Behavioral manifestations of autism in the first year of life. Int J Dev Neurosci.

